# Phosphorus fertilization and maize intercropping with peanut synergistically reshape rhizosphere microbiome and enhance crop yield

**DOI:** 10.3389/fmicb.2025.1732662

**Published:** 2026-01-15

**Authors:** Yan Zheng, Wei Zhao, Xiaona Hu, Zizheng Li, Kaizheng Gao, Nianyuan Jiao

**Affiliations:** College of Agronomy, Henan University of Science and Technology, Luoyang, Henan, China

**Keywords:** Maize||Peanut, microbial community assembly, phosphorus fertilization, rhizosphere microbiome, soilnutrient availability

## Abstract

**Introduction:**

Optimizing nutrient cycling in diversified cropping systems is essential for sustainable agriculture. While intercropping legumes with cereals can enhance complementary resource use, the interaction between phosphorus (P) fertilization and such systems in restructuring rhizosphere microbiomes and driving synergistic productivity gains in alkaline soils remains unclear.

**Methods:**

We conducted a long-term field experiment, integrating amplicon sequencing with comprehensive agronomic and soil analyses to investigate this interaction in a maize-peanut intercropping system under P fertilization.

**Results:**

Phosphorus fertilization significantly increased the yields of intercropped maize (by 52.12%) and peanut (by 43.60%), while simultaneously enhancing the intercropping yield advantage (IYA; +60.77%) and land equivalent ratio (LER; +2.54%). Soil P availability was the dominant environmental driver, explaining 73.46% and 84.39% of the variance in bacterial and fungal community structure, respectively. Phosphorus addition and intercropping selectively enriched keystone functional taxa, including the nitrifying bacterium *Nitrospirae* and the saprophytic fungus *Mortierellomycota*, whose abundances correlated strongly with improved soil nutrient availability and crop performance. Concurrently, intercropping suppressed the pathogen-rich phylum Ascomycota.

**Discussion:**

Our findings demonstrate that the synergy between P fertilization and intercropping enhances crop productivity through a microbiome-mediated mechanism. This synergy restructures the rhizosphere community into a functionally beneficial state, fostering a self-reinforcing plant–microbe–soil feedback loop. This study provides a mechanistic framework for developing integrated, microbiome-informed management strategies to support sustainable agricultural intensification.

## 1 Introduction

Intercropping, the cultivation of two or more crop species in proximity, represents a key strategy for enhancing resource-use efficiency and land productivity compared to conventional monocultures (Ma et al., 2024; Zhang and Li, 2003). By fostering spatial, temporal, and physiological niche differentiation, this practice enables complementary capture of light, water, and nutrients (Brooker et al., 2015). The maize intercropping with peanut (Maize||Peanut) system has attracted considerable scientific attention due to its unique interspecific facilitation (Jiao et al., 2021b). This system exhibits a well-documented reciprocal relationship where both crops mutually enhance nutrient acquisition (e.g., Fe, N) that lead to improved nutrient cycling efficiency (Guo et al., 2014; Hauggaard-Nielsen and Jensen, 2005; Jiao et al., 2021a; Zuo et al., 2000). These beneficial plant–plant interactions contribute to enhanced system productivity while promoting sustainable soil fertility maintenance (Zhang and Li, 2003). The underlying mechanisms involve complex belowground processes mediated by root exudates and microbial activities, which collectively optimize resource utilization in the shared rhizosphere environment.

Phosphorus (P) is a major limiting nutrient in agricultural soils, especially in alkaline or calcareous soils where high pH leads to P fixation and low bioavailability. This limitation significantly impacts agricultural productivity in many regions (Vitousek et al., 2010). In legume-cereal intercropping systems, complementary P-acquisition strategies can improve P-use efficiency. Cereals such as maize may benefit from the rhizosphere acidification induced by legumes like peanut, which increases the solubility of inorganic P (Li et al., 2007). Additionally, legumes can stimulate phosphatase activity and organic acid exudation in the cereal rhizosphere, thereby promoting the mineralization of organic P (Hinsinger et al., 2011; Tang et al., 2021). However, the net effect of these interspecific interactions on soil P cycling, and how it is modulated by P fertilization, remains inadequately understood. This knowledge gap limits our ability to predict and manage P dynamics in legume-cereal intercropping systems.

Intercropping alters the rhizosphere environment, fostering niche differentiation that can reshape soil microbial community composition compared to monocultures (Tian et al., 2019; Wang et al., 2021). Microbial communities are fundamental drivers of soil P cycling, mediating processes such as organic P mineralization, inorganic P solubilization, and P immobilization (Richardson and Simpson, 2011). Specific microbial taxa—including P-solubilizing bacteria (e.g., *Pseudomonas*, *Bacillus*) and fungi (e.g., *Aspergillus*, *Penicillium*), as well as mycorrhizal fungi—play key roles in regulating P availability to plants (Rodríguez and Fraga, 1999; Sharma et al., 2013; Smith and Read, 2010). However, how P fertilization modifies these microbial-mediated processes in legume-cereal intercropping systems, and how these changes feedback to influence P cycling and plant productivity, remains poorly understood, particularly under long-term field conditions.

Based on a long-term field experiment, this study aims to systematically elucidate how phosphorus (P) fertilization and Maize||Peanut intercropping jointly regulate soil microbial communities and crop productivity. We focus on unraveling their individual and interactive effects on rhizosphere microbial diversity and community structure, as well as on the relationships among soil properties, keystone microbial taxa, and crop performance. We hypothesize that P fertilization and intercropping will significantly reshape microbial communities, with fungi showing greater responsiveness than bacteria, and that these practices will selectively enrich microbial taxa involved in key nutrient-cycling processes such as phosphorus solubilization and nitrification. Furthermore, we propose that this microbiome restructuring will be directly linked to enhanced soil phosphorus availability and crop productivity, thereby establishing a self-reinforcing plant–microbe–soil feedback loop. The findings are expected to provide a mechanistic, microbiome-informed foundation for designing integrated management strategies that enhance phosphorus use efficiency and support sustainable agricultural intensification.

## 2 Materials and methods

### 2.1 Field site and experimental design

The long-term P application and Maize||Peanut field experiment commenced in 2010 at the experimental field of Henan University of Science and Technology (34°35′58″ N and 112°24′53″E), located in Luoyang City, Henan Province, China. This region is characterized by a temperate continental monsoon climate, with a mean annual temperature of 13.6 °C, approximately 2060 annual sunshine hours, an average annual precipitation of about 610 mm, and a frost-free period lasting approximately 217 days. The basic physicochemical properties of the 0–20 cm topsoil layer, measured before the experiment initiation, were as follows: 1.32 g·kg^−1^ total nitrogen (TN), 79.90 mg·kg^−1^ available nitrogen (AN), 0.75 g·kg^−1^ total phosphorus (TP), 11.62 mg·kg^−1^ available phosphorus (AP), 223.8 mg·kg^−1^ available potassium (AK), 10.72 g·kg^−1^ soil organic matter (SOM), and a PH of 7.56. The soil is classified as fluvo-aquic and consists of 28% sand, 50% loam, and 22% clay.

The experiment employed a randomized complete block design with three replications, incorporating two factors: phosphorus (P) fertilization level and cropping system. The P factor included two levels: 0 kg P_2_O_5_·ha^−1^ (P0) and 180 kg P_2_O_5_·ha^−1^ (P1), applied annually as a one-time basal dressing using diammonium phosphate before sowing. The cropping system factor comprised three distinct treatments: monocropped maize (SM), monocropped peanut (SP), and Maize||Peanut (MIP); within the intercropping system, the component crops are specifically referred to as intercropped maize (IM) and intercropped peanut (IP) for sampling and analysis. Each plot measured 48 m^2^ (8 m × 6 m). The maize cultivar Zhengdan 958 and peanut cultivar Huayu 16 were used, which were both commonly cultivated by local farmers. In the MIP system, a 2:4 strip configuration was implemented with two rows of maize alternating with four rows of peanut. The planting pattern was as follows: IM with 0.4 m row spacing and 0.2 m plant spacing (density ~50,000 plants·ha^−1^); IP with 0.3 m row spacing and 0.115 m plant spacing (density ~100,000 plants·ha^−1^); and a 0.35 m distance between adjacent maize and peanut rows. For monocultures, SM was planted with 0.6 m row spacing and 0.25 m plant spacing (density ~66,667 plants·ha^−1^), while SP used 0.3 m row spacing and 0.115 m plant spacing (density ~166,667 plants·ha^−1^) (Supplementary Figure S1). All systems received a basal nitrogen application of 90 kg N·ha^−1^ as urea, with an additional 90 kg N·ha^−1^ top-dressed to maize at the pre-tasseling stage. Given the sufficient soil potassium level (223.8 mg AK·kg^−1^), no potassium fertilizer was applied.

### 2.2 Yield measurement and calculation of yield advantages

At harvest, representative 2-m double rows of maize and peanuts were selected from each plot. The air-dried weight of maize grains and peanut pods was measured to calculate their yield. Each treatment was replicated three times. Intercropping yield advantage (IYA) refers to the superior productivity per unit area demonstrated by intercropping systems compared to monocropping systems. The land equivalent ratio (LER) was calculated to evaluate the land-use efficiency of intercropping systems. The LER is defined as the relative land area required by monoculture to produce the same yield as intercropping. A value >1 indicates higher land-use efficiency in intercropping compared to monoculture. The formula is as follows:

$\mathrm{IYA}=(Y_{\mathrm{im}}+Y_{\mathrm{ip}})-(Y_{\mathrm{sm}}\times F_{m}+Y_{\mathrm{sp}}\times F_{p}),$

$\mathrm{LER}=Y_{\mathrm{im}}/Y_{\mathrm{sm}}+Y_{\mathrm{ip}}/Y_{\mathrm{sp}}.$

Where:

IYA represents the intercropping yield advantage (kg·ha^−1^); Y_im_ and Y_ip_ represent the yields of maize and peanuts in the intercropping system (kg·ha^−1^), respectively; Y_sm_ and Y_sp_ represent the yields of monocropped maize and monocropped peanuts (kg·ha^−1^), respectively; F_m_ and F_p_ represent the planting density ratios of intercropped maize and intercropped peanuts, respectively. In this experiment, F_m_ = 0.56 and F_p_ = 0.44 (Chen et al., 2023).

### 2.3 Soil sampling and chemical analysis

Rhizosphere soil sampling was conducted on September 1, 2023, corresponding to the maize milk-ripening and peanut pod-expansion stages. To obtain samples representative of stable interspecific interactions and minimize edge effects, plants were selected from the middle rows of all plots. For the intercropping treatment, sampling targeted the inner rows of both the maize and peanut strips. The sampling procedure was as follows: after clearing surface debris from a 20 × 20 cm area around the plant base, the entire root system was carefully excavated with a sterile shovel. Loosely adhered soil was removed by gentle shaking, and the tightly bound rhizosphere soil (approximately 1 mm layer) was then collected using a sterile brush. The resulting soil samples were passed through a 20-mesh sieve to remove plant debris and other impurities. A total of 24 rhizosphere samples were collected, encompassing the four treatment combinations (SM, SP, IM, IP) under two P levels (P0, P1), with three biological replicates for each combination. Each sample was subsequently divided into two aliquots: one was immediately flash-frozen in liquid nitrogen and stored at −80 °C for molecular microbial analysis, and the other was air-dried for standard soil chemical characterization. The corresponding plant samples were retained for the determination of dry matter biomass and nutrient content.

The soil pH was measured using a PHS-3E pH meter (PHS-3E, China) with a soil-to-water ratio of 1:5 (w/v). Soil available P (AP) was assessed through NaHCO_3_ extraction followed by the molybdenum-antimony resistance colorimetric method (Olsen and Sommers, 1982). Soil available nitrogen (AN) was determined using the alkali-diffusion method. Soil total nitrogen (TN) was extracted with concentrated H_2_SO_4_-H_2_O_2_ and analyzed using a K1305 Kjeldahl nitrogen analyzer (Shanghai Shengsheng Automatic Analysis Instrument Co., LTD., Shanghai). Soil total P (TP) was extracted using H_2_SO_4_-H_2_O_2_ digestion methods, and available iron (AFe) was extracted with the DTPA-TEA method. All extracts were subsequently analyzed using an Agilent 5,110 ICP-OES inductively coupled plasma optical emission spectrometer (Agilent, United States). Soil organic matter (SOM) was determined using the potassium dichromate oxidation method (Walkley and Black, 1934), with results calculated by multiplying the measured organic carbon content by a factor of 1.724.

The maize plants were separated into stems, leaves, husks, cobs, and grains, while the peanut plants were divided into stems, leaves, and pods (kernels + shells). Each treatment was replicated three times. The samples were deactivated at 105 °C for 30 min and then dried at 75 °C until constant weight was achieved. After weighing, the samples were ground into powder (cobs and stems of maize, shells and stems of peanuts were ground separately and then mixed). Plant digestion was carried out using the concentrated H_2_SO_4_-H_2_O_2_ method, and the P concentration was determined by inductively coupled plasma optical emission spectrometry (ICP-OES, Agilent 5,110), N was analyzed using a K1305 Kjeldahl nitrogen analyzer (Shanghai Shengsheng Automatic Analysis Instrument Co., LTD., Shanghai). The P accumulation and N accumulation in each plant was calculated accordingly.

### 2.4 Soil microbial DNA extraction and amplicon sequencing

Soil microbial DNA was extracted using a DNA extraction kit and purified with the E. Z. N. A. Gel Extraction Kit (Omega, United States) from 0.5 g of fresh soil samples. PCR primers 799 F/1193 R (Qiu et al., 2020) and ITS1 F/TS2 R (Schoch et al., 2012) were employed to amplify the bacterial 16S rRNA V5-V7 region and the fungal ITS rRNA ITS1-ITS2 region, respectively. The barcoded amplicons were sequenced on an Illumina Nova 6,000 platform, generating 250 bp paired-end reads at Guangdong Magigene Biotechnology Co., Ltd. (China). After removing the primers, paired-end reads were merged using USEARCH v10 (Edgar, 2010) and subjected to quality control to obtain high-quality Clean Tags using Fastp v0.14.1 (Chen et al., 2018). Subsequently, high-quality clean tags was denoised into amplicon sequence variants (ASVs) by DADA2 (Callahan et al., 2016). For each representative sequence, taxonomic information was annotated using the Unite database (for ITS) (Kõljalg et al., 2013) and the RDP database (for 16S) (Cole et al., 2014) with USEARCH-SINTAX, setting the confidence threshold to ≥0.8 by default. The bacterial ASVs which were annotated as chloroplasts or mitochondria were removed (Xu et al., 2022). Rarefaction curves demonstrated that the sequencing depth was sufficient for the subsequent bacterial diversity analysis (Supplementary Figure S2). Following taxonomic annotation, we identified 17,031 ASVs for bacteria and 5,605 ASVs for fungi from 24 soil samples.

### 2.5 Statistical analyses

All statistical analyses were performed using SPSS version 20.0 (IBM Corp., United States) and R 4.1.0. Three-way analysis of variance (ANOVA) was applied to evaluate the main and interactive effects of P fertilization (P0 vs. P1), crop (maize vs. peanut) and cropping pattern (monocropping vs. intercropping) on soil chemical properties (pH, TN, AN, TP, AP, SOM, and AFe), plant nutrition accumulation (N and P), crop yields, and *α* diversity. Microbial *β*-diversity was assessed using Bray–Curtis dissimilarity matrices analyzed by PERMANOVA (vegan:adonis2, 9,999 permutations) with block stratification, followed by pairwise Adonis tests (Bonferroni-corrected) for significant factors. Analysis of similarities (ANOSIM; vegan:anosim) using Bray-Curtis distances with 9,999 permutations to test global group differences (reported as R statistic: 0 = no separation, 1 = complete separation). Linear Discriminant Analysis Effect Size (LEfSe, LDA score >3.0, α = 0.05) identified differentially abundant taxa across treatments. Environmental drivers were analyzed by redundancy analysis (RDA; vegan:rda) with forward selection (*p* < 0.05), and pairwise associations between microbial taxa and environmental factors were quantified using Spearman correlations. All values are presented as means ± standard deviation (SD) of three biological replicates.

## 3 Results

### 3.1 Soil properties, crop nutrition accumulation, and yield advantages

**Figure 1:**
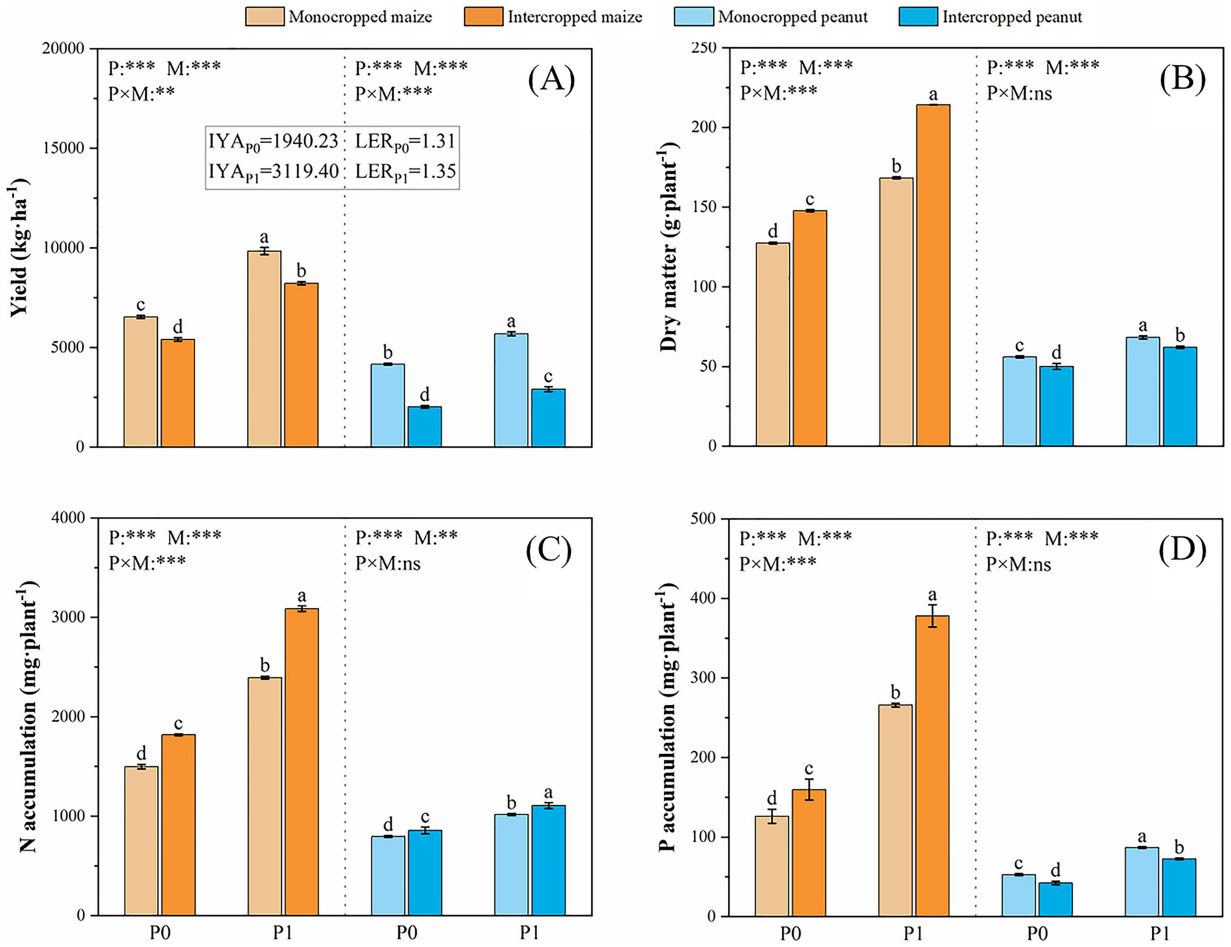
Effects of P fertilization and cropping systems on **(A)** crop yield, **(B)** dry matter accumulation, **(C)** nitrogen (N) accumulation, and **(D)** phosphorus (P) accumulation in Maize||Peanut system. P0 and P1 represent without and with P fertilization, respectively. IYA represents the intercropping yield advantage and LER represents the land equivalent ratio. P represents P fertilizer level effect, M represents cropping pattern effect, and P × M represents the interaction between P level and cropping pattern. Values are means ± SD of three biological replicates. For single-factor ANOVA, different lowercase letters indicate significant differences (*p* < 0.05). For two-factor ANOVA, significance levels are denoted as ***p < 0.001, **p < 0.01, *p < 0.05, ns (not significant).

This study demonstrates that the intercropping system exhibited a significant yield advantage (LER = 1.31–1.35), and P application significantly enhanced system productivity. Specifically, P application increased the yields of intercropped maize and peanut by 52.12 and 43.60%, respectively, compared to the non-P treatment, and further improved the overall IYA and LER by 60.77 and 2.54% (Figure 1A).

Intercropping markedly influenced plant nutrient accumulation and biomass, with effects varying by P availability (Figures 1B–D). Under P-deficient conditions (P0), intercropped maize showed increases of 26.63% in P accumulation, 21.40% in N accumulation, and 15.94% in dry matter relative to monoculture. In contrast, intercropped peanut exhibited reductions of 20.00% in P accumulation and 10.52% in dry matter, although N accumulation increased by 7.60%. When P was applied (P1), these responses were amplified for maize (further increases of 42.19% in P, 28.90% in N, and 27.28% in dry matter). For peanut, P application alleviated the competitive suppression observed under P0: although P accumulation and dry matter remained lower than in monoculture, the reduction magnitudes were smaller (−16.46% vs. −20.00% for P, and −9.06% vs. −10.52% for dry matter), while N accumulation increased further (+8.7% vs. +7.60%). P application (P1) itself dramatically enhanced P and N accumulation across all systems, with the most pronounced increases observed in intercropped maize (136.6 and 69.7% for P and N, respectively).

**Figure 2:**
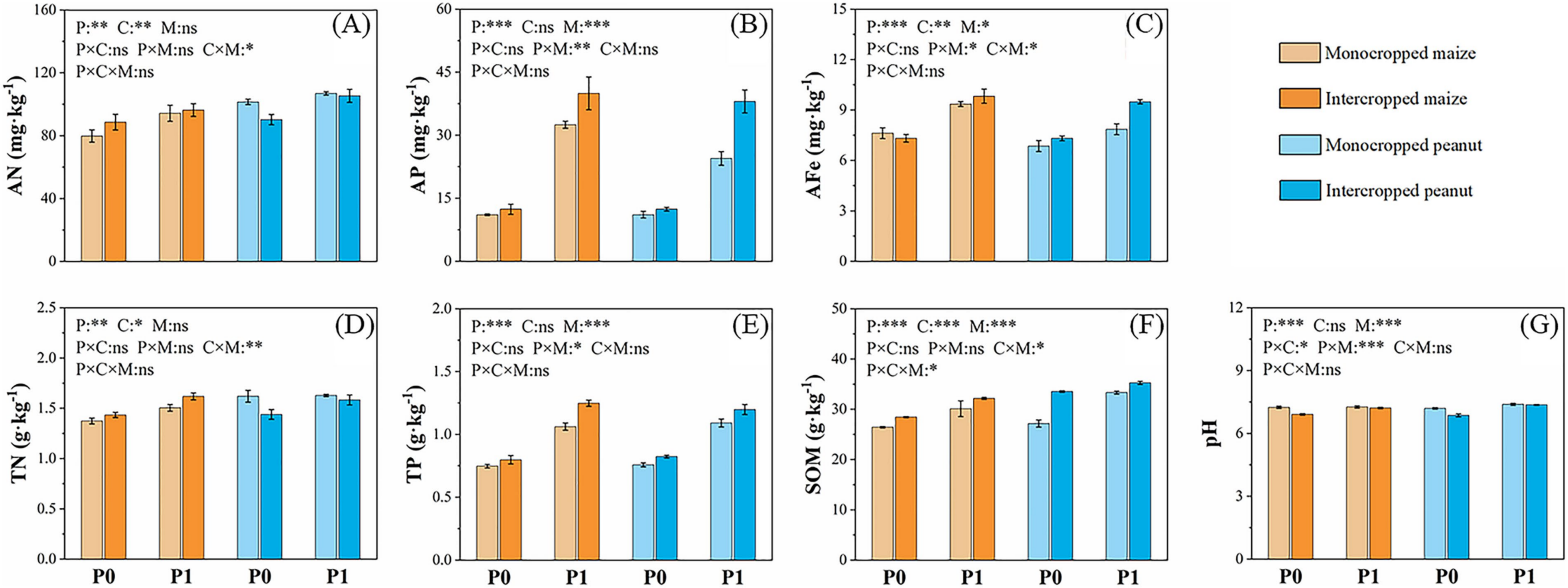
Soil available nitrogen (AN) **(A)**, available phosphorus (AP) **(B)**, available iron (AFe) **(C)**, total nitrogen (TN) **(D)**, total phosphorus (TP) **(E)**, organic matter (SOM) **(F)**, and pH **(G)** in the Maize||Peanut system. P0 and P1 represent without and with P fertilization, respectively. Data are means ± SD (*n* = 3). * *p* < 0.05, ** *p* < 0.01, *** *p* < 0.001, ns (not significant).

Soil chemical properties were more strongly influenced by P application than by cropping pattern (Figure 2). Compared to sole cropping, intercropping significantly increased rhizosphere AP (by 16.9–55.4%) and TP (by 6.7–17.9%) in both crops under P fertilization, while simultaneously reducing soil pH by 0.7–4.7%. However, intercropping effects on N were crop-specific: it increased maize rhizosphere AN by 2.1–11.0% but decreased peanut rhizosphere AN by 1.5–11.1% and TN in both crops by 3.1–11.1%. P application further amplified these trends, elevating intercropped maize AN and TN by 8.7 and 7.5%, and peanut AN and TN by 16.7 and 9.7%, respectively. Soil organic matter (SOM) responded strongly to P addition in the intercropping system, increasing by 9.6% in maize and 29.2% in peanut rhizosphere compared to the non-fertilized condition.

Maize||Peanut strongly promoted maize performance but initially suppressed peanut growth; however, P application crucially alleviated these negative effects on peanut while further enhancing the positive effects on maize. This combination facilitates the mobilization and utilization of key nutrients such as N, P, and Fe in the belowground system, thereby significantly contributing to increased crop yield.

### 3.2 Composition and diversity of soil microbial communities

**Figure 3:**
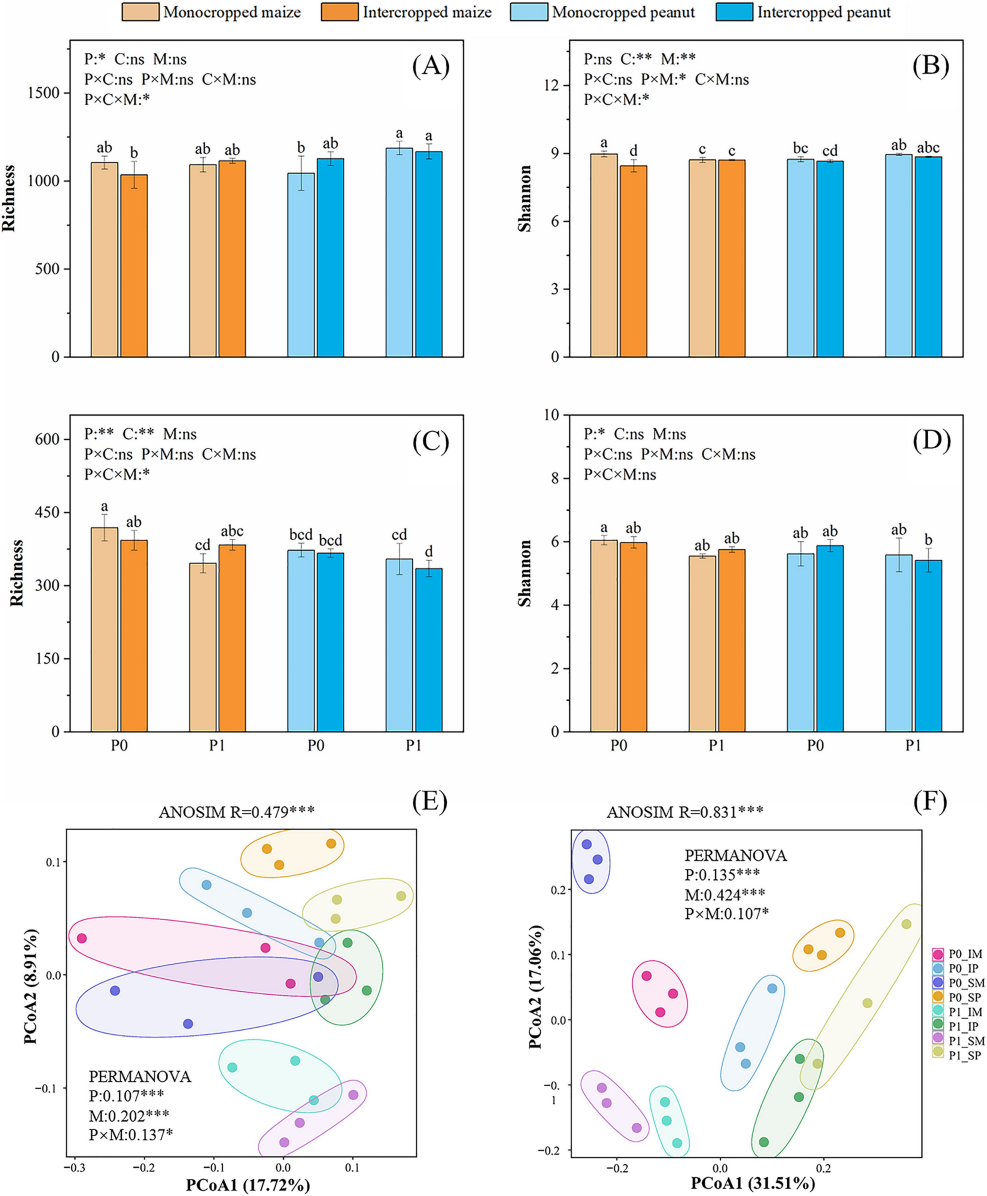
Alpha diversity of soil microbial communities, presented as richness and Shannon index for bacteria **(A,B)** and fungi **(C,D)**. Beta diversity analyzed by principal coordinates analysis (PCoA) based on Bray-Curtis distances for bacterial **(E)** and fungal **(F)** communities at the ASV level. P0 and P1 represent without and with P fertilization, respectively. P represents P fertilizer level effect, C represents crop species effect, M represents cropping pattern effect, and P × C, P × M, C × M, P × C × M represent the interaction between P level, cropping species, and cropping pattern. Values are means ± SD of three biological replicates. For single-factor ANOVA, different lowercase letters indicate significant differences (*p* < 0.05). For three-factor ANOVA, significance levels are denoted as ***p < 0.001, **p < 0.01, *p < 0.05, ns (not significant).

The *α*-diversity of rhizosphere bacterial and fungal communities was assessed using the richness and the Shannon index (Figures 3A–D). P fertilizer significantly influenced bacterial richness, fungal richness, and the Shannon index (*p* < 0.05). Additionally, the cropping pattern had a notable effect on the bacterial Shannon index (*p* < 0.05), while the crop type significantly affected fungal richness (*p* < 0.05). Under the application of P fertilizers, the bacterial richness and Shannon index of soil in the peanut strips were significantly higher than those in the maize strips (*p* < 0.05). Furthermore, the fungal richness and the Shannon index of intercropped maize were greater than those of intercropped peanut, regardless of P fertilization (*p* < 0.05).

Principal Coordinates Analysis (PCoA) and Analysis of Similarities (ANOSIM) based on Bray-Curtis distances confirmed significant structural differences in microbial communities across all treatments (Figures 3E,F; Supplementary Figure S3). Fungal communities exhibited markedly stronger separation between treatments (*R* = 0.831, *p* = 0.001) than their bacterial counterparts (*R* = 0.479, *p* = 0.001). PERMANOVA results demonstrated that cropping pattern was the dominant driver of community variation, explaining 42.4% of fungal community variance (*p* < 0.001) versus 20.2% for bacteria (*p* < 0.001). P application also exerted significantly greater influence on fungal (*R*^2^ = 0.135, *p* < 0.001) than bacterial (*R*^2^ = 0.107, *p* < 0.001) community composition. Notably, we detected significant P × cropping pattern interactions (P × M, *p* < 0.05) in both microbial domains, indicating context-dependent effects of these environmental factors on community assembly. These findings indicate that P application and cropping patterns significantly influence the structure of soil bacterial and fungal communities, and fungal communities demonstrate greater responsiveness to environmental fluctuations compared to bacteria.

**Figure 4:**
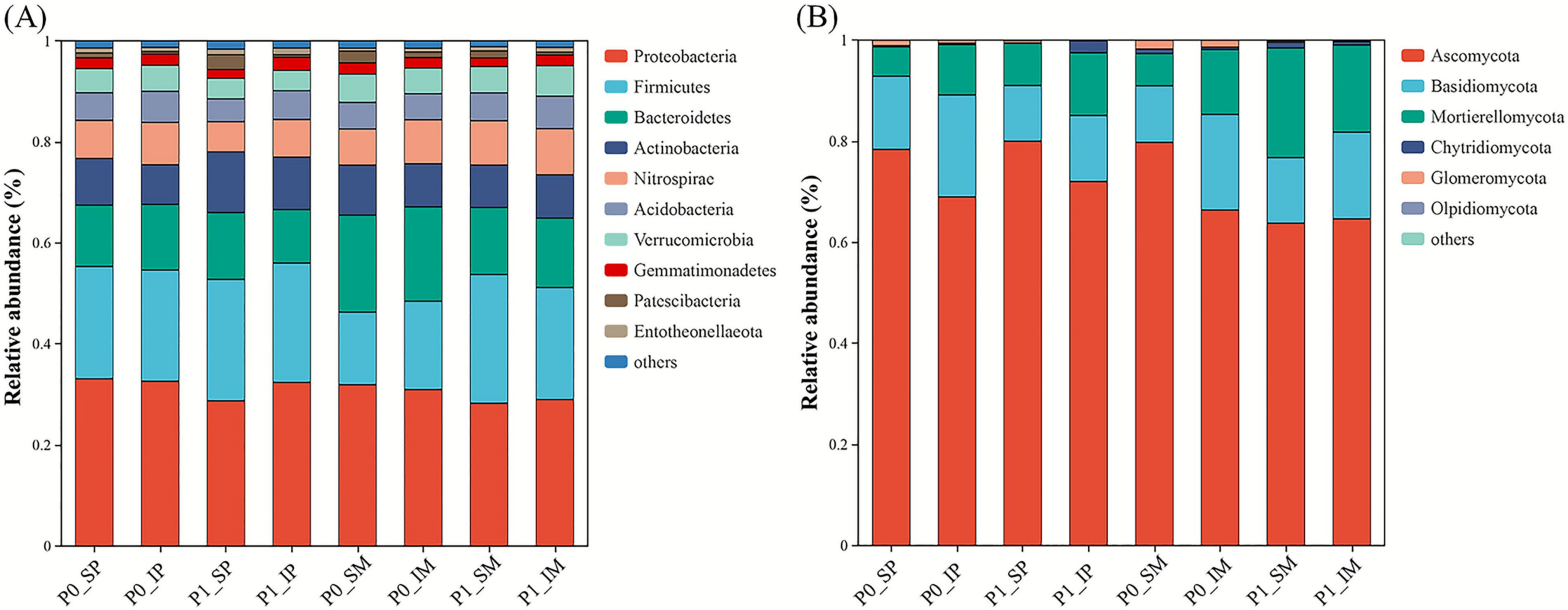
Relative abundance of rhizobacterial **(A)** and rhizofungal **(B)** communities across treatments (phylum). P0 and P1 represent without and with P fertilization, respectively. SM and SP represent monocropped maize and monocropped peanut; IM and IP represent intercropped maize and intercropped peanut, respectively. Values are average relative abundance of three independent biological replicates.

To investigate the effects of P and cropping pattern on the overall structure of the rhizosphere bacterial and fungal community, we analyzed the relative abundances of major bacterial and fungal phyla (Figure 4). The results indicated that the bacterial communities across all treatments were predominantly composed of *Proteobacteria* (28.82–33.91%), *Firmicutes* (14.69–26.04%), *Bacteroidetes* (10.84–19.61%), *Actinobacteria* (7.99–12.33%), and *Nitrospirae* (6.15–9.35%), which together accounted for over 80% of the total bacterial community, forming the core microbiome (Figure 4A; Supplementary Table S1). The three-way ANOVA revealed significant impacts of P application and cropping systems on bacterial phyla. P application markedly increased the relative abundance of *Proteobacteria* (+8.65%, *p* < 0.05) and *Entotheonellaeota* (+37.50%, *p* < 0.01) but decreased *Firmicutes* (−20.18%, *p* < 0.01) in both maize and peanut rhizospheres. Intercropping significantly enhanced the abundance of *Nitrospirae* (+14.86%, *p* < 0.01) and *Gemmatimonadetes* (+15.79%, *p* < 0.05). Furthermore, compared to peanut rhizosphere, maize rhizosphere exhibited higher abundances of *Nitrospirae* (+15.07%, *p* < 0.01), *Bacteroidetes* (+24.69%, *p* < 0.05), and *Verrucomicrobia* (+18.18%, *p* < 0.05), while *Entotheonellaeota* was reduced (−27.27%, *p* < 0.01). In contrast, *Acidobacteria* remained unaffected by P application, cropping system, or their interactions, indicating its stability under the tested conditions.

Fungal communities were dominated by *Ascomycota* (63.77–80.00%), *Basidiomycota* (11.19–20.18%), and *Mortierellomycota* (5.80–21.67%) across all treatments (Figure 4B; Supplementary Table S2). P application increased *Mortierellomycota* abundance (+72.41%, *p* < 0.001) but decreased *Basidiomycota* (−16.05%, *p* < 0.01) and *Glomeromycota* (−83.33%, *p* < 0.01) in both crops. Intercropping stimulated *Mortierellomycota* (+23.58%, *p* < 0.05) and *Basidiomycota* (+39.20%, *p* < 0.001) but suppressed *Ascomycota* (−9.81%, *p* < 0.01). Maize rhizosphere exhibited higher *Mortierellomycota* (+60.44%, *p* < 0.001) but lower *Ascomycota* (−8.29%, *p* < 0.01) compared to peanut rhizosphere. Notably, Chytridiomycota remained unresponsive to all experimental factors, suggesting its resilience to the tested conditions.

### 3.3 Screening for significantly different microorganisms

**Figure 5:**
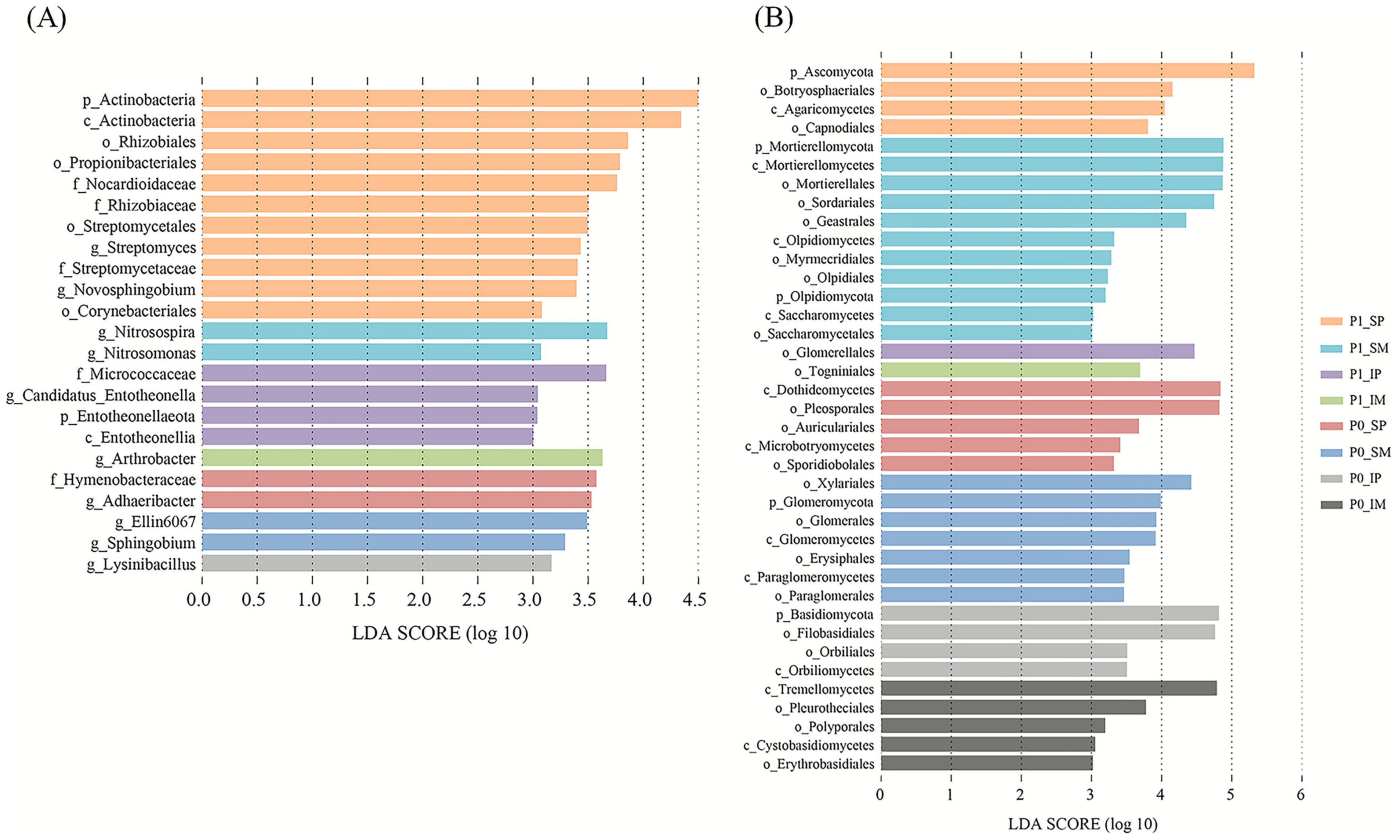
The linear discriminant analysis effect size (LEfSe) analysis of bacterial **(A)** and fungal community **(B)** in the rhizosphere soil with LDA scores higher than 3.0. P0 and P1 represent without and with P fertilization, respectively. SM and SP represent monocropped maize and monocropped peanut; IM and IP represent intercropped maize and intercropped peanut, respectively.

**Figure 6:**
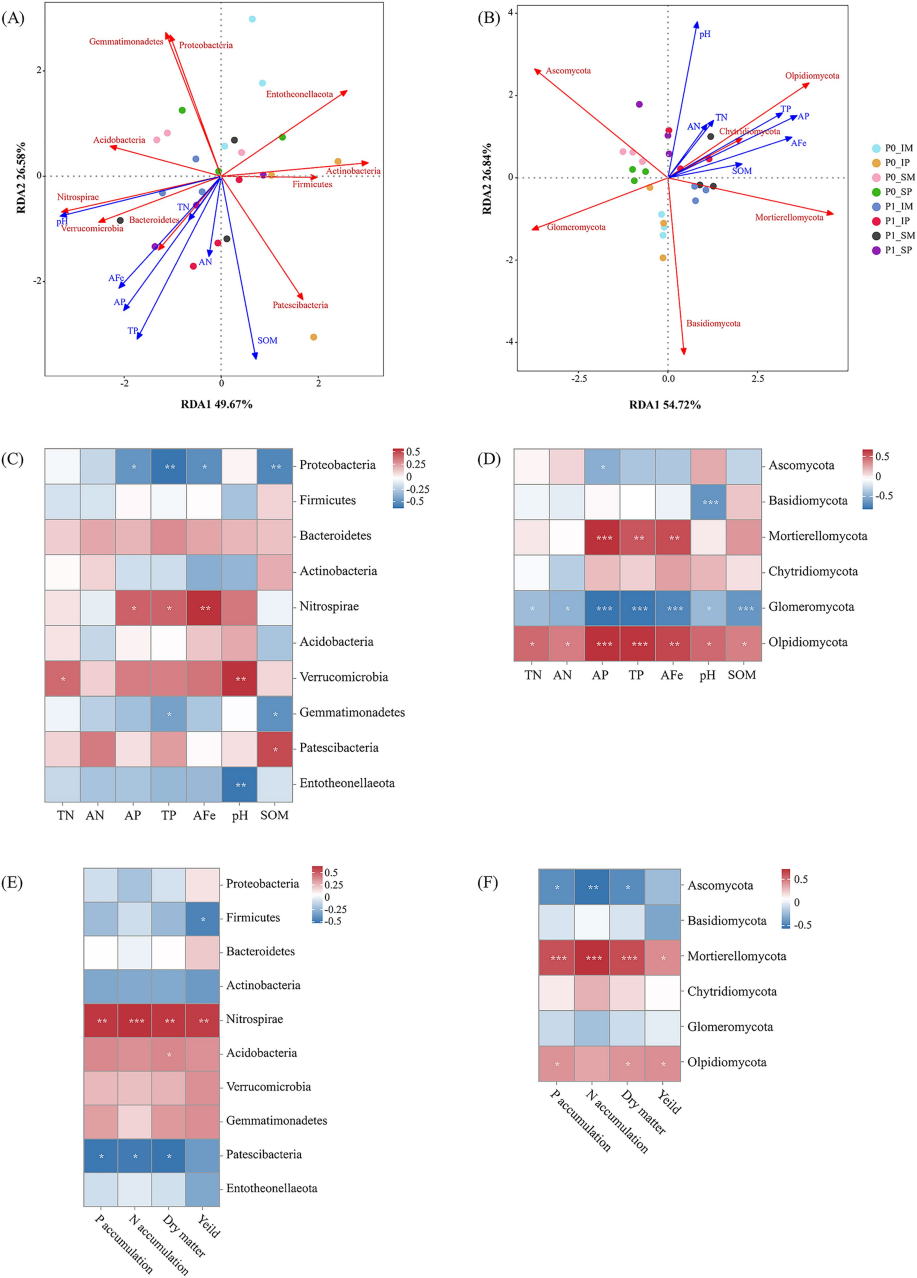
Relationships between soil microbial communities, soil properties, and plant traits. **(A,B)** Redundancy analysis (RDA) of bacterial **(A)** and fungal **(B)** communities constrained by soil properties. **(C,D)** Heatmaps showing Spearman correlations between soil properties and the relative abundance of bacterial **(C)** and fungal **(D)** phyla. **(E,F)** Heatmaps showing Spearman correlations between plant traits and the relative abundance of bacterial **(E)** and fungal **(F)** phyla. For correlation heatmaps **(C–F)**, color gradients denote correlation coefficients, with asterisks indicating statistical significance (*p < 0.05, **p < 0.01, ***p < 0.001). P0 and P1 represent without and with P fertilization, respectively; SM and SP represent monocropped maize and monocropped peanut; IM and IP represent intercropped maize and intercropped peanut, respectively.

LEfSe was employed to identify differentially abundant taxa in the rhizosphere microbiota of maize and peanut monocropped and intercropped crops under both P application and non-application conditions. Given the inherent differences in taxonomic resolution between 16S rRNA and ITS sequencing, bacterial communities were analyzed down to the genus level, while fungal communities were conservatively analyzed to the order level (LDA > 3, *p* < 0.05) (Figure 5). For bacterial communities, the analysis identified 23 microbial biomarkers that were significantly enriched in specific groups, demonstrating distinct group-specific distribution patterns (Figure 6A). The most pronounced enrichment was observed for the phylum *Actinobacteria* and its subordinate taxa, particularly in P applicated monocropped peanut (P1_SP), including the genus *Streptomyces* (LDA = 3.43), the order *Propionibacteriales* (LDA = 3.79), and the order *Rhizobiales* (LDA = 3.86). Monocropped systems, especially under P fertilization (P1_SM), were characterized by an enrichment of ammonia-oxidizing bacteria from the family *Nitrosomonadaceae*, such as *Nitrosospira* (LDA = 3.67) and *Nitrosomonas* (LDA = 3.07). In contrast, the absence of P (P0) selected for different microbial groups, with monocropped peanut (P0_SP) enriching for *Bacteroidetes* members like *Adhaeribacter* (LDA = 3.53) and intercropped peanut (P0_IP) enriching for the firmicute *Lysinibacillus* (LDA = 3.17). Intercropping under P application selected for unique biomarkers in both maize and peanut, namely *Arthrobacter* (P1_IM, LDA = 3.63) and the entire phylum *Entotheonellaeota* (P1_IP, LDA > 3.00), respectively.

LEfSe analysis identified distinct fungal biomarkers under different P management and cropping systems, revealing clear ecological patterning (Figure 6B). P application consistently selected for saprotrophic communities, with the most pronounced response in monocropped maize (P1_SM), where the saprophytic phylum *Mortierellomycota* (LDA > 4.87) and the order *Sordariales* (LDA = 4.75) were strongly enriched, indicating a community specialized in organic matter decomposition. In contrast, P deficiency fostered communities with diverse ecological strategies. Monocropped maize without P (P0_SM) was notably enriched for arbuscular mycorrhizal fungi from the phylum *Glomeromycota* (LDA = 3.99), reflecting a classic symbiotic response to P stress. Concurrently, monocropped peanut without P (P0_SP) was characterized by the enrichment of the class *Dothideomycetes* (LDA = 4.84) and its order *Pleosporales* (LDA = 4.82), which include numerous saprotrophs involved in complex organic matter decomposition. The intercropping systems created unique fungal niches, particularly under P deficiency. Intercropped peanut (P0_IP) and intercropped maize (P0_IM) without P were a key niche for basidiomycetous fungi, showing strong enrichment for the phylum *Basidiomycota* (P0_IP, LDA = 4.82) and the class *Tremellomycetes* (P0_IM, LDA = 4.79), suggesting enhanced decomposition of complex organic compounds. Under P application, intercropped maize (P1_IM) and intercropped peanut (P1_IP) were specifically associated with the orders *Togniniales* (LDA = 3.69) and *Glomerellales* (LDA = 4.47), respectively, demonstrating how intercropping maintains specific fungal signatures even in P-sufficient conditions.

This analysis demonstrates that P availability acts as a primary filter for fungal community assembly, with P deficiency favoring symbiotic and complex organic matter-degrading fungi, while P application selects for rapidly growing saprotrophs. Cropping patterns further refined these communities, with intercropping systems developing distinct fungal profiles potentially contributing to complementary nutrient acquisition strategies.

### 3.4 Relationships between soil chemical properties, plant growth, and microbial communities

RDA identified soil AP and TP as the primary environmental drivers of rhizosphere microbial community structure, with samples clearly separated by P fertilization treatment along the first axis (Figures 6A,B). The P gradient explained a substantial proportion of the variance, particularly for fungi (81.6% on the first two axes), compared to bacteria (76.3%), highlighting the superior sensitivity of fungi to P availability.

At the phylum level, key bacterial and fungal taxa showed distinct and ecologically meaningful correlations with these soil properties and, crucially, with crop performance (Figures 6C–F). Beneficial and nutrient-cycling taxa were positively linked to both soil fertility and plant growth. The nitrifying phylum *Nitrospirae* was positively correlated with soil AFe, AP, and TP. Most importantly, among all bacterial phyla, *Nitrospirae* exhibited the strongest and most significant positive correlations with crop dry matter, N/P accumulation, and yield. Similarly, the saprotrophic phylum *Mortierellomycota* (key for organic matter decomposition) exhibited the strongest positive correlations with soil AFe and AP. Correspondingly, *Mortierellomycota* was the fungal phylum with the strongest positive association with all crop performance metrics. *Olpidiomycota* and *Chytridiomycota* also showed positive associations with plant growth, while *Acidobacteria*, *Verrucomicrobia*, and *Gemmatimonadetes* were positively correlated with plant traits.

In contrast, several taxa were negatively associated with enriched soil conditions and plant productivity. The bacterial phyla *Proteobacteria*, *Firmicutes*, *Bacteroidetes*, *Actinobacteria*, *Entotheonellaeota*, and *Patescibacteria* all showed negative correlations with plant performance, with Patescibacteria displaying the most significant and strongest negative correlation. Among fungi, the phyla *Ascomycota* (which contains many plant pathogens), *Basidiomycota*, and the symbiotic arbuscular mycorrhizal fungi (AMF) phylum *Glomeromycota* were negatively correlated with plant traits, with *Ascomycota* showing the strongest negative association. Many of these taxa (e.g., *Glomeromycota*, *Ascomycota*) also showed strong negative correlations with soil fertility indicators like AFe, AP, and SOM.

## 4 Discussion

### 4.1 Maize||Peanut and P application enhance yield through improved nutrient accumulation and rhizosphere chemistry

This study demonstrates that Maize||Peanut, particularly when combined with P application, synergistically enhances crop productivity and nutrient acquisition despite species-specific responses. P fertilization further strengthens the yield advantage and land equivalent ratio of intercropping, consistent with the characteristic complementary resource use in legume-cereal systems (Ma et al., 2025). Specifically, P application significantly boosts P and N accumulation in intercropped maize (136.63 and 69.67%), likely due to its competitive superiority and potential N transfer from peanut (Zhao F et al., 2022; Zhao X. et al., 2022), whereas intercropped peanut exhibits reduced biomass due to competition for light, water, and nutrients (Zhang et al., 2014). The underground P supplementation effectively alleviates aboveground competition, restructures interspecific relationships, and creates a positive feedback loop that enhances overall yield (Wang et al., 2025).

Intercropping and P fertilization also significantly improved rhizosphere nutrient availability. Available P increased by 16.9–23.0% in maize and 11.8–55.4% in peanut, reflecting complementary mechanisms such as maize-facilitated inorganic P dissolution and peanut-enhanced phosphatase activity (An et al., 2023; Li et al., 2016). P addition further elevated total P in intercropped maize and peanut by 56.25 and 46.34%, confirming improved P-use efficiency (Tang et al., 2021). Crucially, our correlation analyses provide a mechanistic link between these improved soil properties and the observed yield increases. While we did not perform a direct correlation between soil properties and yield, the strong positive associations of key microbial taxa (e.g., *Mortierellomycota*, *Nitrospirae*) with both soil AP/TP levels and crop productivity metrics (Figure 6) indicate that the management-induced enhancements in soil nutrient availability likely translate to yield gains through the assembly of a beneficial, nutrient-cycling microbiome. For instance, the increase in AP was closely linked to the enrichment of *Mortierellomycota*—a saprophytic fungus known for P solubilization (Sang et al., 2022). This same fungal taxon was positively correlated with crop yield. Similarly, the enrichment of the nitrifier *Nitrospirae* in P-fertilized and intercropped treatments links improved N-cycling potential (Daims et al., 2015) with better plant N accumulation and growth. Therefore, the synergy between intercropping and P fertilization appears to enhance yield not merely by directly elevating soil nutrients, but by fostering a microbial community that efficiently mineralizes and cycles these nutrients, thereby making them more bioavailable to plants (Richardson et al., 2009).

Furthermore, intercropping differentially influenced nitrogen dynamics, increasing AN/TN in maize rhizosphere while decreasing it in peanut’s—a pattern consistent with host-specific microbial processing (nitrification in peanut vs. immobilization in maize) (Coskun et al., 2017; Lai et al., 2022). P application amplified these effects, potentially by stimulating biological N fixation (e.g., via enhanced *rhizobial* activity in peanut) in peanut and microbial N turnover (Sun et al., 2020). Intercropping and P addition also elevated soil organic matter (SOM), likely through P-regulated microbial decomposition and root exudation (Dessaux et al., 2016; Li et al., 2023; Sang et al., 2022).

Notably, the classic iron-nitrogen mutualism in this system was further enhanced under P fertilization. Specifically, intercropping combined with P application lowered rhizosphere pH while concurrently increasing AFe content, particularly in the peanut rhizosphere (Jiao et al., 2021a; Zuo et al., 2000). More importantly, P application selectively enriched siderophore-producing bacterial taxa such as *Arthrobacter* (identified in our LEfSe analysis), which synergistically contributes to iron solubilization (Dubinina and Zhdanov, 1975; Orozco-Mosqueda et al., 2013; Sharma et al., 2016). This microbial mediation bridges the P and Fe–N cycles: improved iron nutrition likely promotes symbiotic nitrogen fixation in peanut, and the subsequent increase in nitrogen supply enhances maize growth and potentially its phytosiderophore secretion. In this regulatory loop, P availability acts as a master regulator—by stimulating the activity and abundance of key microbial players, it amplifies the entire plant–microbe-driven Fe–N coupling. Therefore, the observed tripartite interaction (P → Microbiome → Fe–N Coupling) underscores that integrated nutrient management must consider managing the soil microbiome to unlock the full synergistic potential of intercropping systems, especially in soils with co-limiting nutrients.

### 4.2 Maize||Peanut and P application shape rhizosphere microbiome structure and enrich specific functional taxa

Our integrated analysis demonstrates that P application and intercropping collectively reshape the composition, diversity, and functional potential of the rhizosphere microbiome. Soil bacterial and fungal communities distinctly response to P and intercropping. The stronger separation effects observed in fungal communities (ANOSIM *R* = 0.831) compared to bacteria (*R* = 0.479) suggest that fungi are more sensitive to P and intercropping, consistent with previous reports of fungal hyphal networks being highly responsive to environmental changes (Tedersoo et al., 2020). The PERMANOVA results further demonstrate that intercropping was the dominant driver of fungal community structure (*R*^2^ = 0.424), likely due to host-specific associations between plants and fungal taxa (Bulgarelli et al., 2013).

The restructuring of microbial communities was not merely shifts in diversity but also profound changes in taxonomic composition and functional guilds. P availability acted as a powerful environmental filter, triggering a systematic shift in community structure. This was evident in the enrichment of copiotrophic and saprotrophic taxa under P application, such as the bacterial phyla *Proteobacteria*, and the fungal phylum *Mortierellomycota*, which are known for their roles in nutrient mineralization in resource-rich environments (Leff et al., 2015; Lidbury et al., 2022; Sang et al., 2022). Conversely, the dramatic suppression of the symbiotic mycorrhizal phylum *Glomeromycota* under high P conditions illustrates the classic functional trade-off wherein plants reduce carbon allocation to mycorrhizal partners when soil P is readily available (Johnson et al., 2015; Li F. et al., 2020). Furthermore, intercropping significantly enriched nitrifying *Nitrospirae* and *Gemmatimonadetes*, and saprophytic *Mortierellomycota* and *Basidiomycota*, enhancing the microbiome’s capacity for N cycling, P cycling and organic matter decomposition (Baldrian, 2008; Chang et al., 2025; Daims et al., 2015; Kirk and Farrell, 1987; Martinez et al., 2004; Zhi et al., 2023). Concurrently, intercropping suppressed the abundance of Ascomycota—a phylum containing numerous plant pathogens—which may reduce disease pressure and promote overall system health (Dean et al., 2012; Doehlemann et al., 2017; Lv et al., 2020). While, the stability of certain taxa (e.g., *Acidobacteria*, *Chytridiomycota*) suggests functional redundancy or resilience within the microbiome, buffering the ecosystem against drastic perturbations.

The LEfSe results further revealed highly specific biomarker taxa associated with each treatment combination. The enrichment of *Actinobacteria* (e.g., *Streptomycetales*, *Micrococcaceae*) and the nitrifying bacterium *Nitrosospira* in P-fertilized treatments aligns with their known roles in complex organic matter decomposition and ammonia oxidation, respectively. This functional enrichment provides a microbial mechanism for the concurrent increases in soil nitrogen availability (AN, TN) and the enhanced N accumulation observed in crops, particularly in intercropped maize under P addition (Liu et al., 2024). Conversely, the strong association of the symbiotic mycorrhizal phylum *Glomeromycota* with low-P conditions (P0) underscores its ecological role in facilitating plant P acquisition under nutrient stress, which corresponds to the observed strategy of plants relying more on symbiotic pathways when soil P is limiting (Smith and Read, 2010).

Overall, our findings indicate that microbial community assembly in the rhizosphere is governed by a complex interplay of bottom-up (soil nutrient availability) and top-down (plant host selection) processes. These insights highlight the potential of integrated P and intercropping to optimize soil microbial functions and support sustainable agricultural intensification.

### 4.3 P availability drives plant-microbe-soil feedbacks by shaping rhizosphere microbial communities and enhancing crop productivity

To further dissect the environmental drivers and functional outcomes of the restructured microbiome, we examined the specific associations between soil properties, microbial communities, and plant productivity. Our redundancy and correlation analyses reveal that soil P availability (AP and TP) serves as the primary environmental filter associated with the structure of the rhizosphere microbiome, which in turn correlates with plant performance. The high explanatory power of the RDA models (76.25% for bacteria, 81.56% for fungi) along the P gradient underscores the fundamental role of P availability in structuring microbial community assembly (Li Y. et al., 2020).

The strong positive correlations between *Mortierellomycota*, *Nitrospirae*, soil P levels, and plant growth parameters suggest that these taxa may function as keystone microbial indicators of fertile and productive agroecosystems, reflecting their critical roles in nutrient acquisition and plant growth promotion. Conversely, the negative associations between *Patescibacteria*, *Ascomycota*, and plant productivity suggest these taxa may either thrive in nutrient-limited or stressed environments or act as stress-responsive indicators (Dean et al., 2012; Santana-Pereira et al., 2023). Similarly, the negative relationship between *Glomeromycota* (arbuscular mycorrhizal fungi, AMF) and soil P levels aligns with the classic trade-off between AMF symbiosis and direct P availability (Johnson et al., 2015; Treseder and Allen, 2002).

Critically, the strong statistical interconnections between soil nutrients (particularly AP, TP, and SOM), specific microbial taxa, and plant performance suggest the existence of a potential self-reinforcing feedback loop. In this proposed model, P availability favors the establishment of a microbial community that enhances nutrient mineralization, thereby supporting plant growth. Improved plant growth, through root exudation and litter input, can further modify soil chemical properties, creating a positive cycle (Chang et al., 2025; Leff et al., 2015; Zhi et al., 2023). These findings highlight the crucial importance of integrated soil management that simultaneously optimize chemical properties, microbial communities, and plant productivity in agricultural ecosystems.

## 5 Conclusion

This study demonstrates that the synergistic effect of phosphorus fertilization and Maize**||**Peanut intercropping on crop productivity is fundamentally mediated by a reconfigured rhizosphere microbiome. The management practices selectively enriched functional taxa such as *Mortierellomycota* (implicated in phosphorus solubilization) and *Nitrospirae* (key to nitrification), and initiated a reinforcing plant-microbe-soil feedback loop. These findings offer a mechanistic, microbiome-level explanation for enhanced nutrient cycling in diversified cropping systems, while pinpointing specific microbial taxa as promising bioindicators of soil functional status. We acknowledge that the correlative nature of this work highlights associations rather than proves causation; thus, future research employing metagenomics and microbial manipulation is essential to validate causal mechanisms. Nevertheless, this work establishes a concrete ecological framework to guide the development of microbiome-informed management strategies for achieving higher nutrient use efficiency and sustainable agricultural intensification.

## Data Availability

The raw sequencing data generated in this study have been deposited in the NCBI Sequence Read Archive (SRA) under the BioProject accession number PRJNA1354650. The data are publicly accessible via the following direct URL: https://www.ncbi.nlm.nih.gov/bioproject/PRJNA1354650.

## Supplementary material

SUPPLEMENTARY FIGURE S1 Illustration of cropping patterns: **(A)** maize monocropping system, **(B)** peanut monocropping system, and **(C)** Maize||Peanut system.

SUPPLEMENTARY FIGURE S2 Rarefaction curves analysis bacterial communities **(A)** or fungal communities **(B)** The curve was constructed using the Shannon index of ASVs and the number of sequences. P0 and P1 represent without and with P fertilization, respectively. SM and SP represent monocropped maize and monocropped peanut; IM and IP represent intercropped maize and intercropped peanut, respectively.

SUPPLEMENTARY FIGURE S3 Analysis of similarities (ANOSIM) based on Bray-Curtis distances of β-diversity of soil bacteria **(A)** and fungi **(B)** at ASV. P0 and P1 represent without and with P fertilization, respectively. SM and SP represent monocropped maize and monocropped peanut; IM and IP represent intercropped maize and intercropped peanut, respectively.
