# Supplementary figures and images for: Phosphorus fertilization and maize intercropping with peanut synergistically reshape rhizosphere microbiome and enhance crop yield
Source: Front Microbiol. 2026 Jan 15;16:1732662. doi: 10.3389/fmicb.2025.1732662 (PMC12852408; doi:10.3389/fmicb.2025.1732662)

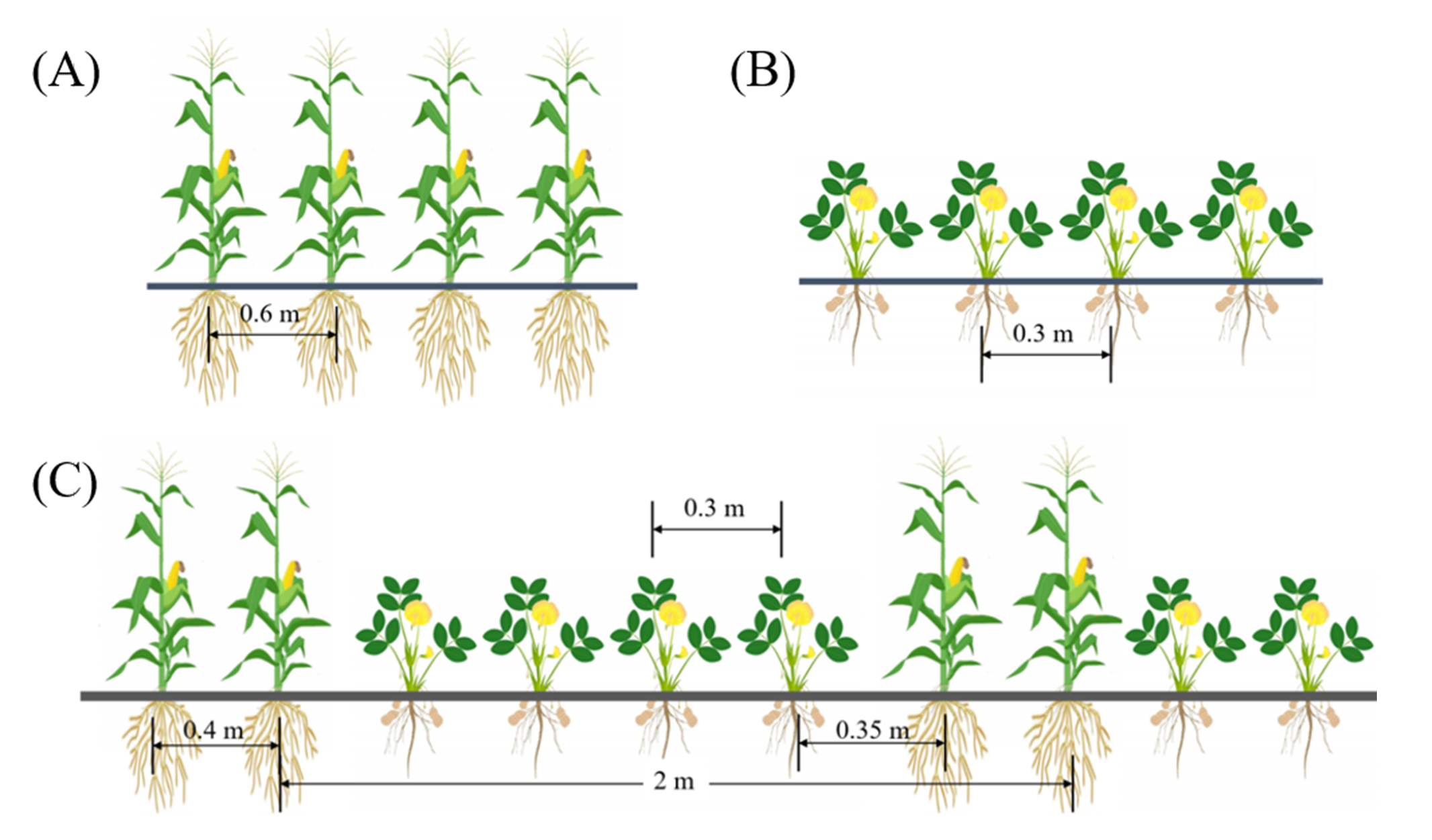

Supplement: SUPPLEMENTARY FIGURE S1 — Illustration of cropping patterns: (A) maize monocropping system, (B) peanut monocropping system, and (C) Maize||Peanut system. [file Image_1.tif]

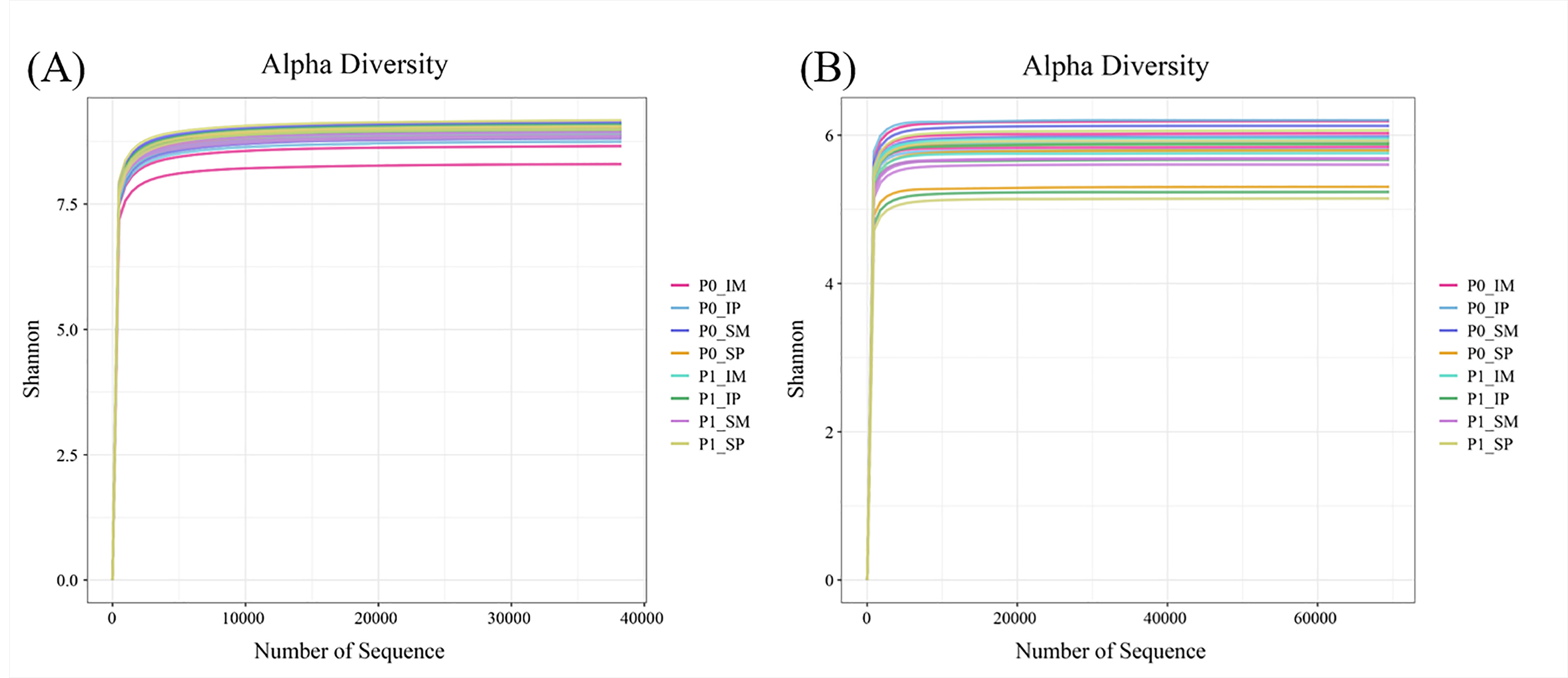

Supplement: SUPPLEMENTARY FIGURE S2 — Rarefaction curves analysis bacterial communities (A) or fungal communities (B) The curve was constructed using the Shannon index of ASVs and the number of sequences. P0 and P1 represent without and with P fertilization, respectively. SM and SP represent monocropped maize and monocropped peanut; IM and IP represent intercropped maize and intercropped peanut, respectively. [file Image_2.tif]

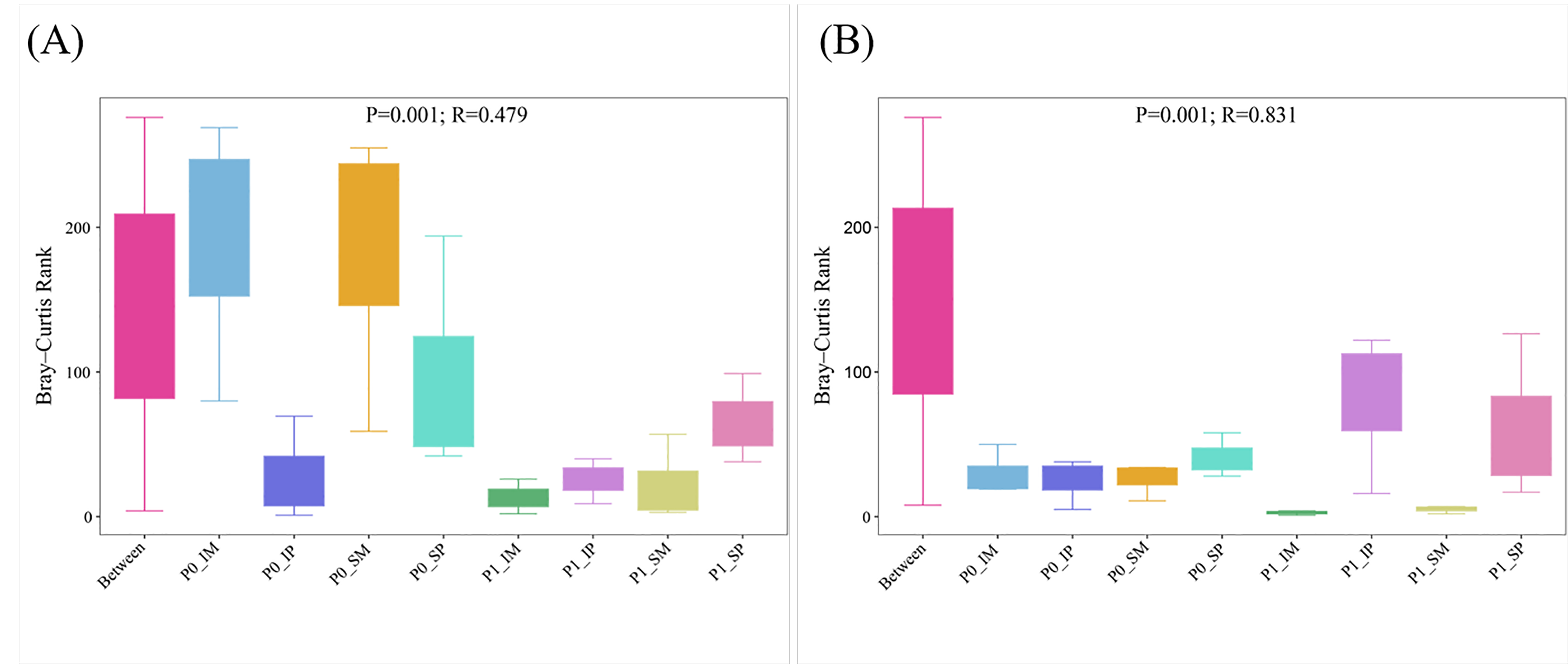

Supplement: SUPPLEMENTARY FIGURE S3 — Analysis of similarities (ANOSIM) based on Bray-Curtis distances of β-diversity of soil bacteria (A) and fungi (B) at ASV. P0 and P1 represent without and with P fertilization, respectively. SM and SP represent monocropped maize and monocropped peanut; IM and IP represent intercropped maize and intercropped peanut, respectively. [file Image_3.tif]
