# Supplementary material for: Phosphorus fertilization and maize intercropping with peanut synergistically reshape rhizosphere microbiome and enhance crop yield
Source: Front Microbiol. 2026 Jan 15;16:1732662. doi: 10.3389/fmicb.2025.1732662 (PMC12852408; doi:10.3389/fmicb.2025.1732662)
Supplement: Supplementary file 4 [file Table_1.docx]

**Table S1.** Results of three-way ANOVA showing the effects of phosphorus level (P), crop species (C), and cropping pattern (M) on the relative abundance of dominant bacterial phyla.

| **Phylum** | **Source of variation** | **df** | **F** | ***P-value*** |  |
| --- | --- | --- | --- | --- | --- |
| *Proteobacteria* | P level (P) | 1 | 5.799 | **0.028** |  |
|  | Crop species (C) | 1 | 2.507 | 0.133 |  |
|  | Cropping Pattern (M) | 1 | 0.476 | 0.500 |  |
|  | P × C | 1 | 0.062 | 0.807 |  |
|  | P × M | 1 | 1.886 | 0.189 |  |
|  | C × M | 1 | 0.640 | 0.436 |  |
|  | P × C × M | 1 | 0.334 | 0.572 |  |
| *Firmicutes* | P level (P) | 1 | 9.193 | **0.008** |  |
|  | Crop species (C) | 1 | 3.796 | 0.069 |  |
|  | Cropping Pattern (M) | 1 | 0.016 | 0.901 |  |
|  | P × C | 1 | 3.805 | 0.069 |  |
|  | P × M | 1 | 1.098 | 0.310 |  |
|  | C × M | 1 | 0.006 | 0.942 |  |
|  | P × C × M | 1 | 0.991 | 0.334 |  |
| *Bacteroidetes* | P level (P) | 1 | 3.077 | 0.099 |  |
|  | Crop species (C) | 1 | 5.176 | **0.037** |  |
|  | Cropping Pattern (M) | 1 | 0.075 | 0.788 |  |
|  | P × C | 1 | 1.840 | 0.194 |  |
|  | P × M | 1 | 0.130 | 0.723 |  |
|  | C × M | 1 | 0.062 | 0.806 |  |
|  | P × C × M | 1 | 0.434 | 0.520 |  |
| *Actinobacteria* | P level (P) | 1 | 2.491 | 0.134 |  |
|  | Crop species (C) | 1 | 2.700 | 0.120 |  |
|  | Cropping Pattern (M) | 1 | 2.808 | 0.113 |  |
|  | P × C | 1 | 7.599 | **0.014** |  |
|  | P × M | 1 | 0.305 | 0.589 |  |
|  | C × M | 1 | 0.533 | 0.476 |  |
|  | P × C × M | 1 | 0.479 | 0.499 |  |
| *Nitrospirae* | P level (P) | 1 | 0.099 | 0.757 |  |
|  | Crop species (C) | 1 | 10.548 | **0.005** |  |
|  | Cropping Pattern (M) | 1 | 9.665 | **0.007** |  |
|  | P × C | 1 | 11.334 | **0.004** |  |
|  | P × M | 1 | 0.178 | 0.679 |  |
|  | C × M | 1 | 0.077 | 0.786 |  |
|  | P × C × M | 1 | 1.837 | 0.194 |  |
| *Acidobacteria* | P level (P) | 1 | 0.003 | 0.957 |  |
|  | Crop species (C) | 1 | 0.138 | 0.715 |  |
|  | Cropping Pattern (M) | 1 | 3.535 | 0.078 |  |
|  | P × C | 1 | 4.343 | 0.054 |  |
|  | P × M | 1 | 1.156 | 0.298 |  |
|  | C × M | 1 | 0.595 | 0.452 |  |
|  | P × C × M | 1 | 0.152 | 0.701 |  |
| *Verrucomicrobia* | P level (P) | 1 | 0.434 | 0.520 |  |
|  | Crop species (C) | 1 | 4.643 | **0.047** |  |
|  | Cropping Pattern (M) | 1 | 0.126 | 0.728 |  |
|  | P × C | 1 | 1.735 | 0.206 |  |
|  | P × M | 1 | 0.232 | 0.637 |  |
|  | C × M | 1 | 0.001 | 0.979 |  |
|  | P × C × M | 1 | 1.089 | 0.312 |  |
| *Gemmatimonadetes* | P level (P) | 1 | 1.667 | 0.215 |  |
|  | Crop species (C) | 1 | 1.817 | 0.196 |  |
|  | Cropping Pattern (M) | 1 | 7.785 | **0.013** |  |
|  | P × C | 1 | 0.994 | 0.334 |  |
|  | P × M | 1 | 11.084 | **0.004** |  |
|  | C × M | 1 | 2.757 | 0.116 |  |
|  | P × C × M | 1 | 0.664 | 0.427 |  |
| *Patescibacteria* | P level (P) | 1 | 0.232 | 0.636 |  |
|  | Crop species (C) | 1 | 0.232 | 0.637 |  |
|  | Cropping Pattern (M) | 1 | 21.901 | **0.000** |  |
|  | P × C | 1 | 10.965 | **0.004** |  |
|  | P × M | 1 | 2.107 | 0.166 |  |
|  | C × M | 1 | 0.768 | 0.394 |  |
|  | P × C × M | 1 | 5.211 | **0.036** |  |
| *Entotheonellaeota* | P level (P) | 1 | 9.277 | **0.008** |  |
|  | Crop species (C) | 1 | 11.419 | **0.004** |  |
|  | Cropping Pattern (M) | 1 | 1.104 | 0.309 |  |
|  | P × C | 1 | 0.125 | 0.728 |  |
|  | P × M | 1 | 1.003 | 0.332 |  |
|  | C × M | 1 | 0.184 | 0.674 |  |
|  | P × C × M | 1 | 0.933 | 0.349 |  |
| Note: Significance level was set at α = 0.05. Significant p-values are in bold. | | | | |  |
|  |  |  |  |  |  |
