# Supplementary material for: Phosphorus fertilization and maize intercropping with peanut synergistically reshape rhizosphere microbiome and enhance crop yield
Source: Front Microbiol. 2026 Jan 15;16:1732662. doi: 10.3389/fmicb.2025.1732662 (PMC12852408; doi:10.3389/fmicb.2025.1732662)
Supplement: Supplementary file 5 [file Table_2.docx]

**Table S2.** Results of three-way ANOVA showing the effects of phosphorus level (P), crop species (C), and cropping pattern (M) on the relative abundance of dominant fungal phyla.

| **Phylum** | **Source of variation** | **df** | **F** | ***P-value*** |
| --- | --- | --- | --- | --- |
| *Ascomycota* | P level (P) | 1 | 3.760 | 0.070 |
|  | Crop species (C) | 1 | 12.850 | **0.002** |
|  | Cropping Pattern (M) | 1 | 18.156 | **0.001** |
|  | P × C | 1 | 10.215 | **0.006** |
|  | P × M | 1 | 5.260 | **0.036** |
|  | C × M | 1 | 0.465 | 0.505 |
|  | P × C × M | 1 | 3.659 | 0.074 |
| *Basidiomycota* | P level (P) | 1 | 10.139 | **0.006** |
|  | Crop species (C) | 1 | 0.176 | 0.681 |
|  | Cropping Pattern (M) | 1 | 35.365 | **0.000** |
|  | P × C | 1 | 10.434 | **0.005** |
|  | P × M | 1 | 4.762 | **0.044** |
|  | C × M | 1 | 1.612 | 0.222 |
|  | P × C × M | 1 | 0.001 | 0.986 |
| *Mortierellomycota* | P level (P) | 1 | 38.569 | **0.000** |
|  | Crop species (C) | 1 | 29.218 | **0.000** |
|  | Cropping Pattern (M) | 1 | 6.272 | **0.023** |
|  | P × C | 1 | 13.076 | **0.002** |
|  | P × M | 1 | 7.979 | **0.012** |
|  | C × M | 1 | 2.360 | 0.144 |
|  | P × C × M | 1 | 7.423 | **0.015** |
| *Chytridiomycota* | P level (P) | 1 | 1.487 | 0.240 |
|  | Crop species (C) | 1 | 0.013 | 0.911 |
|  | Cropping Pattern (M) | 1 | 0.551 | 0.469 |
|  | P × C | 1 | 0.677 | 0.423 |
|  | P × M | 1 | 1.412 | 0.252 |
|  | C × M | 1 | 2.847 | 0.111 |
|  | P × C × M | 1 | 1.477 | 0.242 |
| *Glomeromycota* | P level (P) | 1 | 17.990 | **0.001** |
|  | Crop species (C) | 1 | 2.311 | 0.148 |
|  | Cropping Pattern (M) | 1 | 2.246 | 0.153 |
|  | P × C | 1 | 2.607 | 0.126 |
|  | P × M | 1 | 0.189 | 0.670 |
|  | C × M | 1 | 0.395 | 0.539 |
|  | P × C × M | 1 | 0.019 | 0.892 |
| *Olpidiomycota* | P level (P) | 1 | 52.191 | **0.000** |
|  | Crop species (C) | 1 | 1.753 | 0.204 |
|  | Cropping Pattern (M) | 1 | 2.842 | 0.111 |
|  | P × C | 1 | 2.550 | 0.130 |
|  | P × M | 1 | 2.782 | 0.115 |
|  | C × M | 1 | 0.261 | 0.617 |
|  | P × C × M | 1 | 0.006 | 0.937 |

Note: Significance level was set at α = 0.05. Significant p-values are in bold.
